# Elevated Blood Lead Levels of Children in Guiyu, an Electronic Waste Recycling Town in China

**DOI:** 10.1289/ehp.9697

**Published:** 2007-03-28

**Authors:** Xia Huo, Lin Peng, Xijin Xu, Liangkai Zheng, Bo Qiu, Zongli Qi, Bao Zhang, Dai Han, Zhongxian Piao

**Affiliations:** Central Laboratory and the Key Immunopathology Laboratory of Guangdong Province, Shantou University Medical College, Shantou, China

**Keywords:** children, China, environmental, e-waste, Guiyu, lead

## Abstract

**Background:**

Electronic waste (e-waste) recycling has remained primitive in Guiyu, China, and thus may contribute to the elevation of blood lead levels (BLLs) in children living in the local environment.

**Objectives:**

We compared the BLLs in children living in the e-waste recycling town of Guiyu with those living in the neighboring town of Chendian.

**Methods:**

We observed the processing of e-waste recycling in Guiyu and studied BLLs in a cluster sample of 226 children < 6 years of age who lived in Guiyu and Chendian. BLLs were determined with atomic absorption spectrophotometry. Hemoglobin (Hgb) and physical indexes (height and weight, head and chest circumferences) were also measured.

**Results:**

BLLs in 165 children of Guiyu ranged from 4.40 to 32.67 μg/dL with a mean of 15.3 μg/dL, whereas BLLs in 61 children of Chendian were from 4.09 to 23.10 μg/dL with a mean of 9.94 μg/dL. Statistical analyses showed that children living in Guiyu had significantly higher BLLs compared with those living in Chendian (*p* < 0.01). Of children in Guiyu, 81.8% (135 of 165) had BLLs > 10 μg/dL, compared with 37.7% of children (23 of 61) in Chendian (*p* < 0.01). In addition, we observed a significant increasing trend in BLLs with increasing age in Guiyu (*p* < 0.01). It appeared that there was correlation between the BLLs in children and numbers of e-waste workshops. However, no significant difference in Hgb level or physical indexes was found between the two towns.

**Conclusions:**

The primitive e-waste recycling activities may contribute to the elevated BLLs in children living in Guiyu.

Disposal of electronic waste, or e-waste, is an emerging global environmental issue, as these wastes have become the most rapidly growing segment of the municipal waste stream in the world [Dahl 2002; Halluite et al. 2005; Jang and Townsend 2003; Schmidt 2002; Silicon Valley Toxics Coalition (SVTC) 2001]. It is reported that approximately 500 million computers became obsolete between 1997 and 2007 in the United States (National Safety Council 1999). Up to 80% of e-waste from the United States has seeped into Asia and Africa (Johnson 2006; Puckett et al. 2002; Schmidt 2002, 2006; SVTC 2001). It is noteworthy that the United States is the only developed country today that has not ratified the United Nations Basel Convention, which bans the export of hazardous wastes to developing countries (United Nations Environment Programme 1992, 2006; USA Today 2002).

Together with New Delhi in India, Guiyu in Shantou, Guangdong Province, China (Figure 1), is one of the popular destinations of e-waste (Brigden et al. 2005; Puckett et al. 2002). Within a total area of 52 km^2^ and local population of 132,000 (in 2003), Guiyu has accommodated millions of tons of e-waste from overseas and domestic a year. Nearly 60–80% of families in the town have engaged in e-waste recycling operations conducted by small scale family-run workshops, with approximately 100,000 migrant workers employed in processing e-waste. Because the implementation of a clean and safe high-tech recovery process was very expensive (Allsopp et al. 2006), the processes and techniques used during the recycling activities in Guiyu were very primitive. The result was that many tons of e-waste material and process residues were dumped in workshops, yards, roadsides, open fields, irrigation canals, riverbanks, ponds, and rivers. Hazardous chemicals can be released from e-wastes through disposal or recycling processes, threatening the health of local residents. Several studies have reported the soaring levels of toxic heavy metals and organic contaminants in samples of dust, soil, river sediment, surface water, and groundwater of Guiyu (Brigden et al. 2005; Puckett et al. 2002; Wang and Guo 2006; Wang et al. 2005; Wong et al. 2006; Yu et al. 2006). Previously, we have shown that the residents in Guiyu had high incidence of skin damage, headaches, vertigo, nausea, chronic gastritis, and gastric and duodenal ulcers, all of which may be caused by the primitive recycling processing of e-waste (Qiu et al. 2004).

Of many toxic heavy metals, lead is the most widely used in electronic devices for various purposes, resulting in a variety of health hazards due to environmental contamination (Jang and Townsend 2003; Musson et al. 2006; Vann et al. 2006). Lead enters biological systems via food, water, air, and soil. Children are particularly vulnerable to lead poisoning—more so than adults because they absorb more lead from their environments (Baghurst et al. 1992; Grigg 2004; Guilarte et al. 2003; Jain and Hu 2006; Needleman 2004; Safi et al. 2006; Wasserman et al. 1998). The U.S. Centers for Disease Control and Prevention (CDC) defined elevated blood lead levels (BLLs) as those ≥ 10 μg/dL in children ≤ 6 years of age (CDC 1991). Nevertheless, studies have increasingly shown that low blood lead concentrations, even < 10 μg/dL, were inversely associated with children’s IQ scores and academic skills (Canfield et al. 2003; Lanphear et al. 2000, 2005; Nevin 2000; Schnaas et al. 2006). Therefore, no safety margin at existing exposures has been identified (Chiodo et al. 2004; Koller et al. 2004).

Considering the potential heavy metal contamination in the local living environment of Guiyu, we hypothesized that children living in Guiyu may have elevated BLLs and thus their physical and mental development may have been affected. In this study, we evaluated the mean BLLs in children 1–6 years of age living in Guiyu and compared them with those living in the neighboring town of Chendian, where no e-waste processing was taken.

## Materials and Methods

### Geographic location and site description

**Figure 1:**
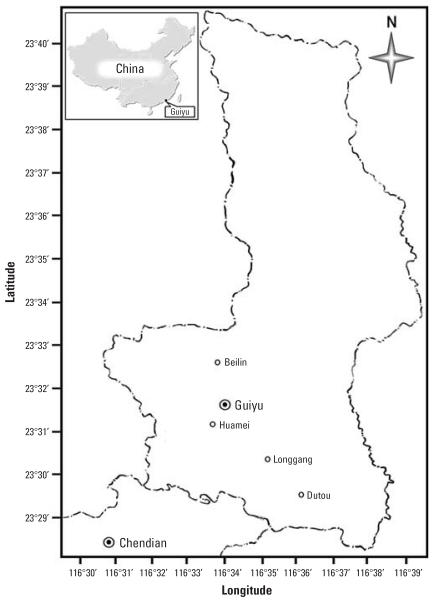
Map of Guiyu and Chendian, with latitude and longitude.

There are 28 villages with a total area of 52 km^2^ and a resident population of 132,000 and around 100,000 migrant workers in Guiyu (Figure 1). We chose four villages for their differences in the scale and type of e-waste processing. Beilin village has dense e-waste workshops mainly involved in equipment dismantling, circuit board baking, and acid baths; Dutou village specializes in plastics sorting, including manually stripping plastic materials from electronic products and then crudely classifying them; Huamei village had workshops similar to those of Beilin, but they are fewer and scattered; and Longgang village was involved in plastic reprocessing in which plastics collected from Dutou and other villages were washed and smashed into tiny pieces of recycled plastic. We used the neighboring town of Chendian as a control because the local residents work mainly in the textiles industry, not in e-waste processing. The population, traffic density, lifestyle, and socioeconomic status were very similar to those of Guiyu.

### Study population

The study population was composed of children ≤ 6 years of age. No children involved in the study had any occupational exposure to e-waste. A cluster sample of 165 children with a median age of 5.0 years lived in the four villages of Guiyu (Figure 1). Sixty-one children with a median age of 4.0 years resided in Chendian were included in the study for comparison. After written informed consent was obtained from the parents or guardians, blood samples were collected from the children at village kindergartens. To facilitate the counseling process, advice on dietary and eating habits to minimize lead exposure were provided to the local residents. All children found to have high BLLs were advised to get further hospital treatment. The study was approved by the Human Ethics Committee of Shantou University Medical College.

### Measurement of BLLs and hemoglobin

Venipuncture blood samples were obtained from each volunteer at the kindergarten, and collected in lead-free tubes by trained nurses. Lead in total blood was analyzed by graphite furnace atomic absorption spectrometry (GFAAS), which consisted of a Shimadzu AA-660 AAS and GFA-4B graphite furnace atomizer and an ACS-60G autosampler (Shimadzu Corporation, Kyoto, Japan). The main parameters used for the determination were a wavelength of 283.3 nm, current of 8 mA, a slit width of 1.00 nm, drying at 150°C, ashing at 325°C, and atomization at 1,400°C. The accuracy of the method was controlled by recoveries between 95% and 107% from the spiked blood samples. Repeated analyses of standard solutions confirmed the method’s precision. The BLLs were expressed in micrograms per deciliter (1 μg/dL = 0.0484 μmol/L). Meanwhile, we assessed hemoglobin (Hgb) levels by hemoglobin cyanide method with hemoglobinometer (XK-2, JiangSu, China).

### Evaluation of physical developmental indexes

Children’s physical growth and development, such as body height, weight, and head and chest circumferences were measured when blood samples were collected. Weight and height were measured using a weighing and height scale (TZ120; Yuyao Balance Instrument Factory, Yuyao, China) with maximum weight of 120 kg (minimum scale, 50 g) and minimum height of 70 cm (minimum scale, 0.5 cm). Head and chest circumferences were measured using graduated anthropometric tapes.

### Statistical analyses

We performed statistical analyses using SPSS version 10.0 software (SPSS, Chicago, IL, USA). We used independent sample *t*-tests or covariance analyses for comparisons of mean, chi-square analyses for test of frequency data, and linear regression analysis for the association between BLLs and age. Differences were considered significant with a *p*-value < 0.05.

## Results

### Observation of e-waste processing

**Figure 2:**
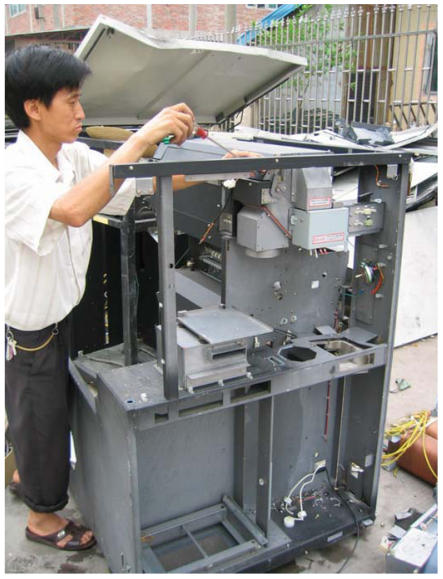
Equipment dismantled with simple tools.

**Figure 3:**
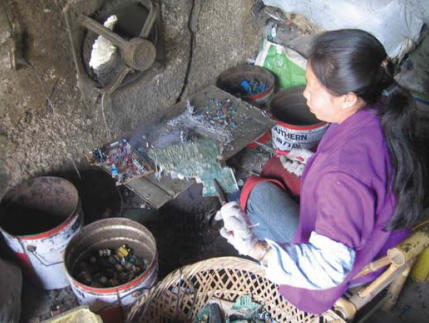
Circuit board baking.

**Figure 4:**
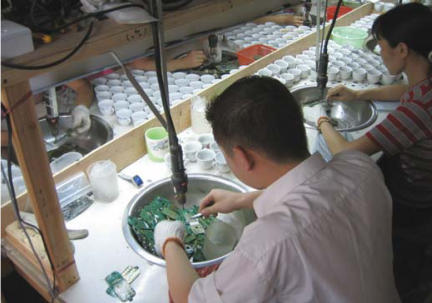
Circuit boards of cell phone being dismantled.

**Figure 5:**
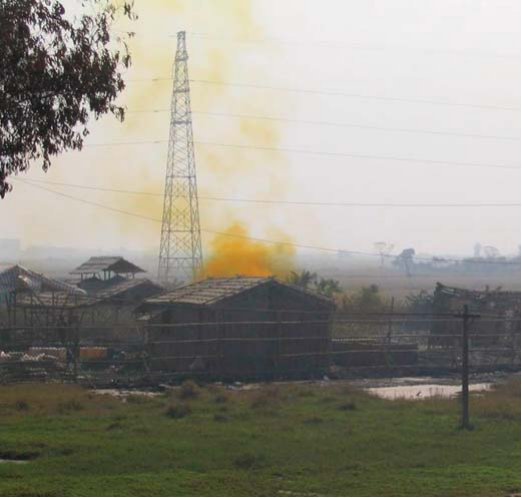
Yellow smoke from acid bath hut.

**Figure 6:**
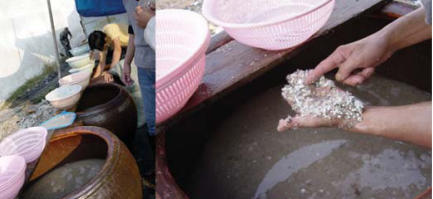
Plastic reprocessing by gravitational separation into ceramic jugs with brine.

The primitive e-waste recycling procedures in Guiyu were mainly as follows: *a*) Old electronic equipment was dismantled (Figure 2) with electric drill, cutter, hammer, and screwdriver into component parts such as monitor, hard drive, CD driver, wires, cables, circuit boards, transformer, charger, battery, and plastic or metal frame that are sold for reuse or to other workshops for further recycling. *b*) Circuit boards (Figure 3) of computers and other large appliances were heated over coal fires to melt the solder to release valuable electronic components, such as diodes, resistors, and microchips. *c*) Circuit boards of cell phones and other hand-held devices were taken apart by a electrothermal machine (Figure 4), which was a particular environmental and human health concern in the processing of e-waste in Guiyu. *d*) In acid baths (Figure 5), some microchips and computer parts were soaked to extract precious gold and palladium, from which the waste acids were discharged into nearby fields and streams. *e*) Wires and cables were stripped or simply burnt in open air to recover metals. *f*) Printer cartridges were ripped apart for their toner and recyclable aluminum, steel, and plastic parts. *g*) Plastic [e.g., polyvinyl chloride (PVC), acrylonitrile butadiene styrene copolymer (ABS), high-density polyethylene (HDPE)] was sorted by workers according to rigidity, color, and luster. Plastic scraps that cannot be sorted visually must be burned and classified by burning odor. Another way to sort different plastics was gravitational separation into ceramic jugs with brine (Figure 6), after which the pieces were spread on the sidewalk to dry; *h*) For reprocessing, after sorting plastic scraps were fed into grinders that spit out tiny pieces of plastic. *i*) For metals sorting and reprocessing, transformers, chargers, batteries, and cathode-ray tubes were separated and hammered open for recycling metals such as copper, steel, silver, aluminum, which were then reprocessed to raw material.

Although the methods for processing e-waste were primitive, the coordination of e-waste recycling in Guiyu was very well organized into specific tasks. Workshops specializing in dismantled equipment would not conduct circuit board baking or plastics and metals reprocessing. The chain of recycling components from each type of e-waste was well established in the town.

### BLLs in children

We collected blood from 165 children in Guiyu and 61 children in Chendian and measured the BLLs in these children. Table 1 shows that the BLLs corresponded to the children’s age, sex, and town of residence. As expected, BLLs among Guiyu children were much higher than those in the children of Chendian (*p* < 0.01). Among Guiyu children, 135 (81.8%) had BLLs > 10 μg/dL, whereas 23 (37.7%) in Chendian (*p* < 0.01) had high levels. Among 135 (81.8%) Guiyu children with elevated BLLs, 61.8% and 20% had BLLs > 10 μg/dL and 20 μg/dL respectively, but lead levels > 45 μg/dL were not found. And BLLs of Guiyu increased somewhat with age (*p* < 0.01); older children tended to have higher BLLs than younger ones. We found no evidence for the association in lead concentrations or prevalence of elevated BLLs differentiated by sex (both *p >* 0.05).

Table 2 presents BLLs for 165 exposed children in the four villages. The findings showed that BLLs from different villages were in the following descending order: Beilin, 19.34 μg/dL > Dutou, 17.86 μg/dL > Huamei, 14.23 μg/dL > Longgang, 13.13 μg/dL (Table 2). Children living in Beilin, where the number of e-waste workshops specializing in equipment dismantling, circuit board baking, and acid baths, had the highest BLLs. Dutou, which had many workshops specializing in plastics sorting, including strip plastic materials from e-waste, had the second highest BLLs in children. Huamei had e-waste workshops similar to those of Beilin, but fewer and less centralized; the BLLs of Huamei children were much lower than those of Beilin and Dutou. Longgang, a village specializing in reprocessing plastics collected from other villages that had no workshops directly processing e-waste, had the lowest BLLs. There was a significant difference in BLLs among the children of the four villages (*p* < 0.01). In Beilin and Dutou, 88.8% and 100% children had elevated BLLs > 10 μg/dL, respectively.

As far as physical indexes and Hgb levels were concerned, there was no significant difference between Guiyu and Chendian (*p* > 0.05, Table 3).

## Discussion

In this study, we observed that the processing of e-waste in Guiyu was very primitive and the recycling industry depended mainly on manual processing methods. Despite the fact that the coordination of the e-waste recycling is well organized in family-based small business units, the manual processing methods and the deposition of the e-waste have contributed to the contamination by heavy metals in the living environment. Examination of the possible impact of the e-waste industry on the BLLs of children living in Guiyu revealed that Guiyu children had significantly higher BLLs than Chendian children. Of children tested in Guiyu, 81.8% had BLLs > 10 μg/dL, indicating a correlation between the BLLs in children and the numbers of e-waste workshops. We speculated that the elevated BLLs in Guiyu children may be directly caused by the contamination of the lead during e-waste recycling. However, further study should be conducted to determine the relationship between BLLs in Children and the actual lead contamination in the environment.

Lead is considered one of the major heavy metal contaminants during the process of e-waste recycling. A cathode ray tube inside a television set or a computer monitor contains an average of 4–8 lb lead; monitor glass contains about 20% lead by weight; a typical battery weighs 36 lb and contains about 18 lb of lead. For decades, lead as a major component of solders has been used to attach electronic components to printed circuit boards. Lead compounds have also been used as stabilizers in some PVC cables and other products. Our study demonstrated in Guiyu a significant increasing trend in BLLs with increasing age; older children tended to have higher BLLs than younger ones. This might be the result of increasing exposure risk because older children might have more outdoor activities. In addition, it may also be attributed to the fact that the heaviest lead-contaminated zone in air after the burning of the e-waste was 75–100 cm above the ground (Wang and Zhang 2006), which was the height range for normal Chinese children 5–6 years of age.

In China, the mean BLL of children was 9.29 μg/dL, and 33.8% of the subjects had BLLs > 10 μg/dL; boys’ mean BLL was 9.64 μg/dL, significantly higher than the girls’ mean BLL of 8.94 μg/dL (*p* < 0.001) (Wang and Zhang 2006). Generally in China, BLLs of children living in industrial and urban areas were significantly higher than those of children in suburbs and rural areas (Wang and Zhang 2006). In Guiyu, the BLLs of children were higher than the mean level in China, and there were no significant different between boys and girls. Although Guiyu is rural, the children’s BLLs were nearly double those of a nearby urban area, Shantou City (7.9 μg/dL; Luo et al. 2003). Compared with results from studies conducted in some other part of Guangdong province, such as Zhongshan City (7.45 μg/dL; Huang et al. 2003) and Shenzhen City (9.06 μg/dL; Wang et al. 2003), we observed higher BLLs not only in Guiyu children, but also in Chendian children (9.94 μg/dL). The lead contamination may have spread from Guiyu to nearby Chendian by dust, river, and air and contributed to the elevation of Chendian children’s BLLs.

In conclusion, elevated BLLs in Guiyu children are common as a result of exposure to lead contamination caused by primitive e-waste recycling activities. Lead contamination from e-waste processing appears to have reached the level considered to be a serious threat to children’s health around the e-waste recycling area. Based on these threats, it is necessary to increase public awareness about the effects of exposure to lead from e-waste and arouse local governments’ interest in public health and safety, so that an infrastructure for safe management of e-waste can be established. More important, responsible management strategies should be undertaken to minimize e-waste production and make e-waste components more easily recycled and reused.

## Correction

In the Abstract and Discussion, the percentage of Guiyu children with BLLs > 10 μg/dL has been corrected from 88% in the original manuscript published online to 81.8%.

## Figures and Tables

**Table 1:** Children’s BLLs (μg/dL) in Guiyu and Chendian.

	Guiyu				Chendian			
Characteristic	No.	Mean ± SD	Range	No. (%) ≥ 10 μg/dL	No.	Mean ± SD	Range	No. (%) ≥ 10 μg/dL
Total	165	15.30 ± 5.79	4.40–32.67	135 (81.8)	61	9.94 ± 4.05	4.09–23.10	23 (37.7)
Age (years)								
1–4	22	12.88 ± 4.88	4.40–22.09	16 (72.7)	14	10.02 ± 3.03	5.72–14.23	6 (42.9)
4–5	49	14.01 ± 6.06	4.90–30.25	35 (71.4)	20	9.46 ± 3.82	4.09–23.10	5 (25.0)
5–6	94	16.54 ± 5.56	4.50–32.67	84 (89.4)	27	10.27 ± 4.73	4.52–22.18	12 (44.4)
Sex								
Male	87	15.14 ± 5.91	4.40–32.67	71 (81.6)	34	10.63 ± 4.30	6.01–23.10	16 (47.1)
Female	78	15.48 ± 5.68	5.21–29.49	64 (82.1)	27	9.09 ± 3.61	4.09–20.91	8 (29.6)

**Table 2:** BLLs for exposed children (*n* = 165) in four villages of Guiyu.

BLLs	Beilin	Dutou	Huamei	Longgang
No.	18	41	48	58
Range (μg/dL)	9.16–30.25	11.07–26.37	4.40–29.50	6.32–32.67
Mean^a^ ± SE (μg/dL)	19.34 ± 1.26	17.86 ± 0.86	14.23 ± 0.80	13.13 ± 0.69
4.40–9.99 μg/dL [no. (%)]	2 (11.1)	0 (0)	14 (29.2)	14 (24.1)
10.00–19.99 μg/dL [no. (%)]	8 (44.4)	26 (63.4)	28 (58.3)	40 (69.0)
20.00–32.70 μg/dL [no. (%)]	8 (44.4)	15 (36.6)	6 (12.5)	4 (6.9)

a Mean adjusted by age.

**Table 3:** Characteristics of the study population, in Guiyu and Chendian.

	Guiyu		Chendian	
Characteristic	Range	Mean ± SE	Range	Mean ± SE
Height (cm)				
Male	86.00–115.50	103.67 ± 0.49^a^	92.80–115.60	102.90 ± 0.79^a^
Female	69.50–113.70	102.66 ± 0.49^a^	86.80–110.90	101.81 ± 0.84^a^
Weight (kg)				
Male	11.00–33.50	15.74 ± 0.27^a^	13.60–20.90	16.22 ± 0.43^a^
Female	7.10–19.20	15.09 ± 0.18^a^	12.50–19.10	15.61 ± 0.31^a^
Head circumference (cm)				
Male	44.00–54.00	49.51 ± 0.16^a^	46.00–52.80	49.22 ± 0.25^a^
Female	42.80–51.50	48.41 ± 0.16^a^	44.50–49.90	47.60 ± 0.27^a^
Chest circumference (cm)				
Male	45.20–75.50	51.58 ± 0.36^a^	48.70–62.30	52.95 ± 0.58^a^
Female	42.20–55.20	51.12 ± 0.24^a^	48.00–55.20	51.08 ± 0.42^a^
Hgb level (g/L)	93–165	127.55 ± 1.31	95–161	123.46 ± 2.25

a Mean adjusted by age.
